# Supplementary material for: The interactome of intact mitochondria by cross-linking mass spectrometry provides evidence for coexisting respiratory supercomplexes
Source: Mol Cell Proteomics. 2017 Dec 8;17(2):216–32. doi: 10.1074/mcp.RA117.000470 (PMC5795388; doi:10.1074/mcp.RA117.000470)
Supplement: Supplemental Data [file supp_RA117.000470_133922_0_supp_23978_fzffwf.zip › spectra_annotation/mito_spectra_annotation/80-1-6-1-17-1.pdf]

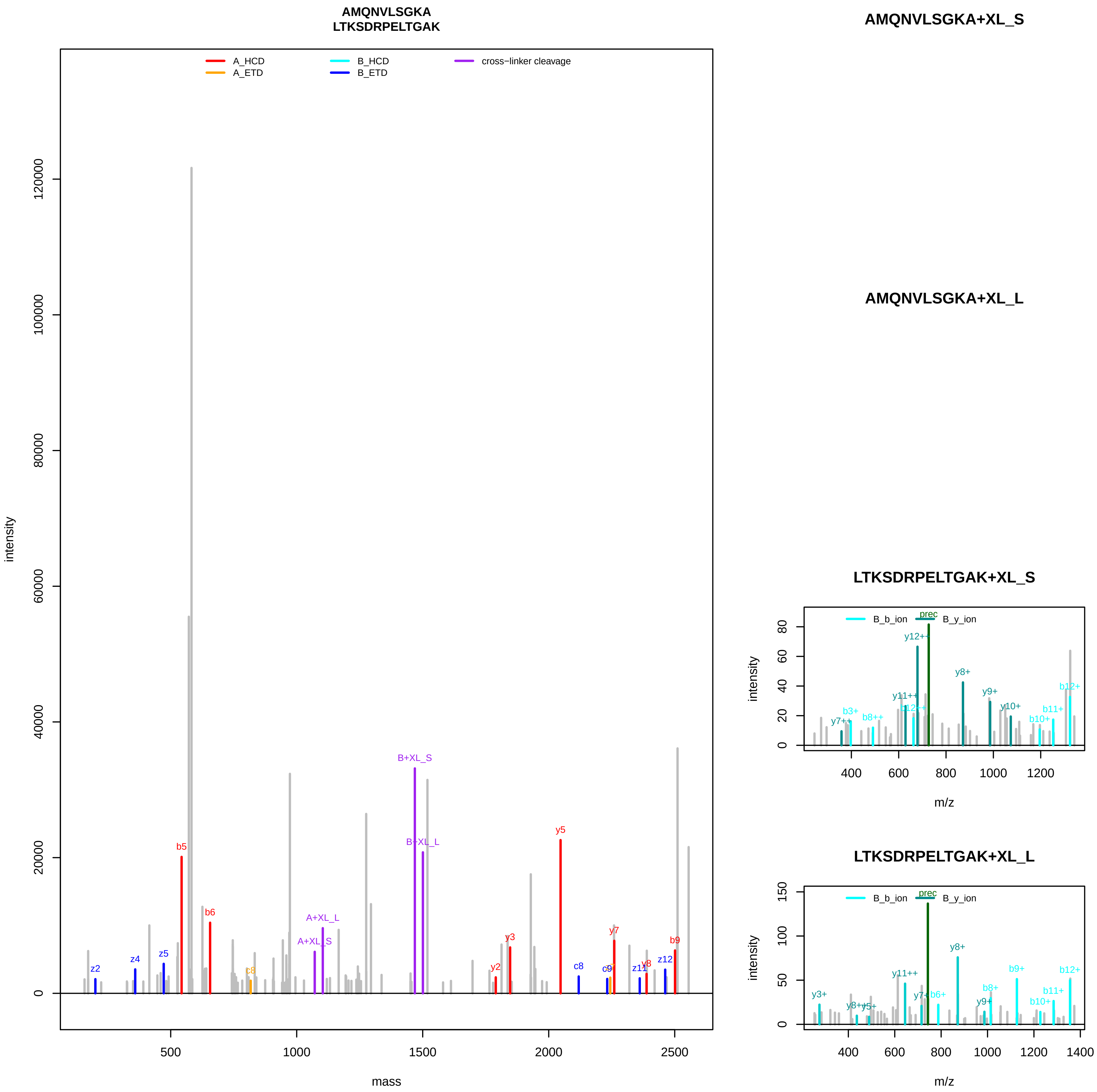

AMQNVLSGKA
LTKSDRPELTGAK
A_HCD
A_ETD
B_HCD
B_ETD
cross−linker cleavage
intensity
mass
B+XL_S
B+XL_L
A+XL_L
A+XL_S
b5
b6
y5
y3
y2
y7
y8
b9
z2
z4
z5
c8
c9
z11
z12
AMQNVLSGKA+XL_S
AMQNVLSGKA+XL_L
LTKSDRPELTGAK+XL_S
B_b_ion
B_y_ion
prec
y12++
y8+
y9+
y10+
y11++
b12++
b3+
b8++
y7++
b10+
b11+
b12+
m/z
LTKSDRPELTGAK+XL_L
y3+
y8++
y5+
y7+
b6+
b8+
b9+
y9+
b10+
